# Iatrogenic calcinosis cutis in 9-month-old baby boy: a case report

**DOI:** 10.1186/s13256-022-03306-w

**Published:** 2022-03-01

**Authors:** Eman Ahmed Alghaith, Ghada Abdullah AlQahtani, Jamal Ahmed Omer

**Affiliations:** King Fahad Medical City, Riyadh, Saudi Arabia

**Keywords:** Iatrogenic calcinosis cutis, Intravenous calcium treatment, Hand swelling, Calcification

## Abstract

**Background:**

Calcinosis cutis is a rare condition, characterized by an accumulation of calcium salts in the skin and subcutaneous tissue. There are several types of this condition, including dystrophic, metastatic, idiopathic, calciphylaxis, and iatrogenic calcinosis cutis. The type related to our case is iatrogenic calcinosis cutis, and one its possible causes is calcium intravenous infusion. Physicians should be aware of this condition when giving calcium infusion.

**Case presentation:**

Here we report the case of a 9-month-old Arabic - Saudi baby boy, who presented with abnormal movement for 1 day. Upon further investigation, his abnormal movement was found to be a manifestation of hypocalcemia and vitamin D deficiency. He was treated with intravenous calcium gluconate. Later, he had a treatment-related complication of intravenous calcium at the site of venipuncture causing swelling, which was initially soft but progressed to hard, over the left hand. Eventually, he was diagnosed with a case of iatrogenic calcinosis cutis due to intravenous calcium treatment.

**Conclusion:**

There are multiple differential diagnoses of calcinosis cutis, as it resembles many other conditions. Careful history-taking, physical examination, and other investigations, such as radiological investigations, will aid in reaching a more accurate diagnosis and, thus, early treatment and intervention. Frequently checking the intravenous line and diluting the intravenous calcium may help reduce the occurrence of iatrogenic calcinosis cutis.

## Introduction

Calcinosis cutis is a condition in which there is an accumulation of calcium salts in subcutaneous tissues. One of its causes is treatment with intravenous calcium gluconate, calcium chloride, for diseases associated with hypocalcemia [1]. Lesions and nodules in calcinosis cutis can form slowly and gradually or they can be progressive and severe [1]. Clinical presentation can vary from affecting limited areas to lesions affecting large areas [2]. In the case of calcinosis cutis caused by treatment with intravenous calcium, lesions usually appear at the site of venipuncture [1]. Diagnosis and management of calcinosis cutis can be challenging as it resembles many other conditions. Moreover, there are other types of calcinosis cutis, including dystrophic, metastatic, idiopathic, and calciphylaxis [1]. Here, we report the case of a 9-month-old baby boy who presented with calcinosis cutis of left hand following the treatment of hypocalcemic seizure 2 weeks earlier with calcium gluconate infusion.

## Case presentation

A 9-month-old boy, Arabic - Saudi, known to have mild eczema, presented to the emergency department with a history of left-hand swelling that had started 5 days before the presentation. It started on the dorsal aspect of his left hand and extended above the wrist joint. It was associated with tenderness and was warm to the touch. He was afebrile, and all other joints were unaffected. The swelling affected his daily activities. There was a pigmented area on his left hand with no itchiness, pus discharge, or skin changes. Systemic review was unremarkable. His neonatal history was unremarkable with uneventful pregnancy. He has no family history of similar condition, no history of consanguinity between parents, and no previous history of congenital or metabolic diseases in the family. He was admitted 1 month before this presentation as a case of hypocalcemic seizure where he was treated with intravenous calcium gluconate on the same side as the swelling.

**Fig. 1:**
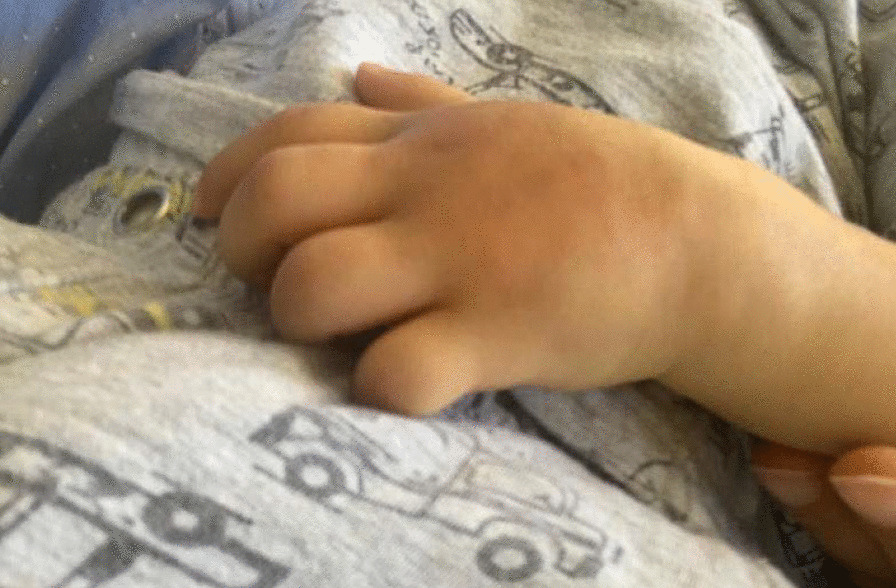
Swollen dorsal aspect of the left hand with discoloration

Local examination (Fig. 1) showed a swollen left hand, mainly in the dorsal aspect, crossing the wrist joint, with bluish discoloration, redness, hotness, and tenderness, no fluctuation or skin changes or discharge, and restrictive hand motion with good and intact pulses.

Investigations were done, with hematology (complete blood count: white blood cells 11.3 × 10^9^/L, hemoglobin 12.7 g/dL, platelets 530 × 10^9^/L), inflammatory markers [erythrocyte sedimentation rate (ESR) 9 mm/hour, C-reactive protein (CRP) 0.36 mg/L], and biochemistry (sodium 139 mmol/L, potassium 4.7 mmol/L, urea 1.5 mmol/L, creatinine 14 μmol/L, corrected calcium 2.5 mmol/L, phosphate 1.8 mmol/L, vitamin D 120.6 nmol/L) were all within normal range. A diagnosis of post-intravenous-line cellulitis was made, and he was admitted for clindamycin intravenous antibiotic treatment. Upon admission, left-hand ultrasound was done, and it was normal. The swelling started to subside slowly; he was discharged with oral Augmentin, to be followed up in the outpatient clinic in a week. On follow-up, he was doing well, with a normal bone profile and biochemistry. Examination of the hand (Fig. 1) revealed hard swelling over the dorsal aspect of the left hand with mild restriction of range of motion. X-ray was ordered during this visit.

**Fig. 2:**
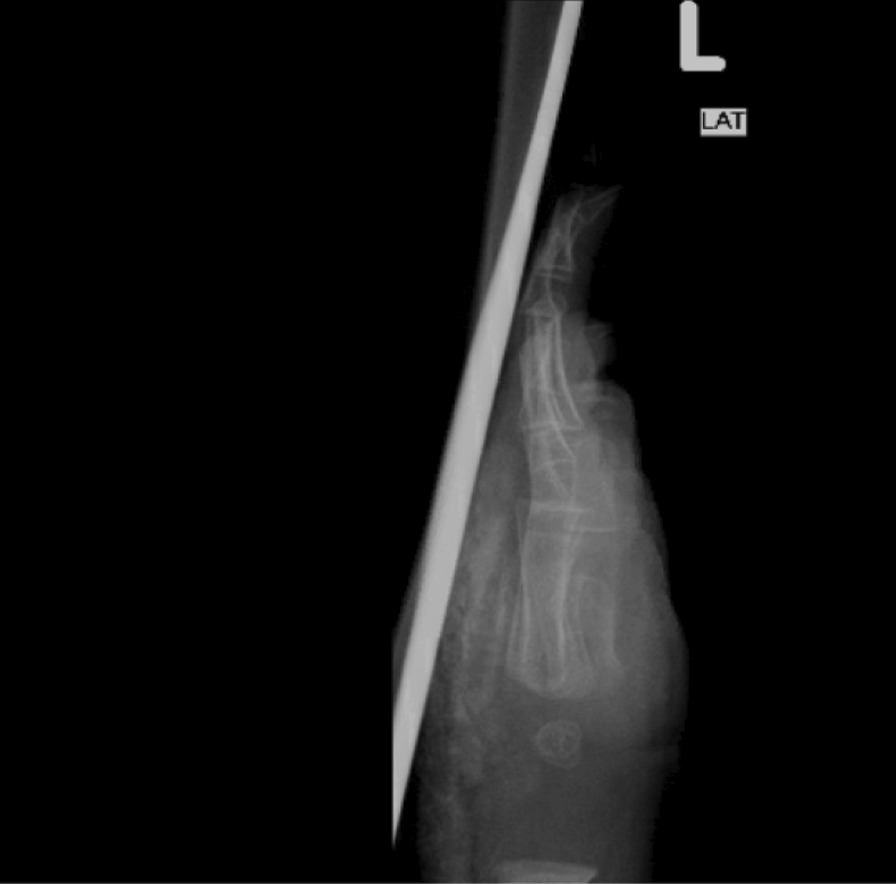
Subcutaneous calcification of the dorsal aspect of the left hand

**Fig. 3:**
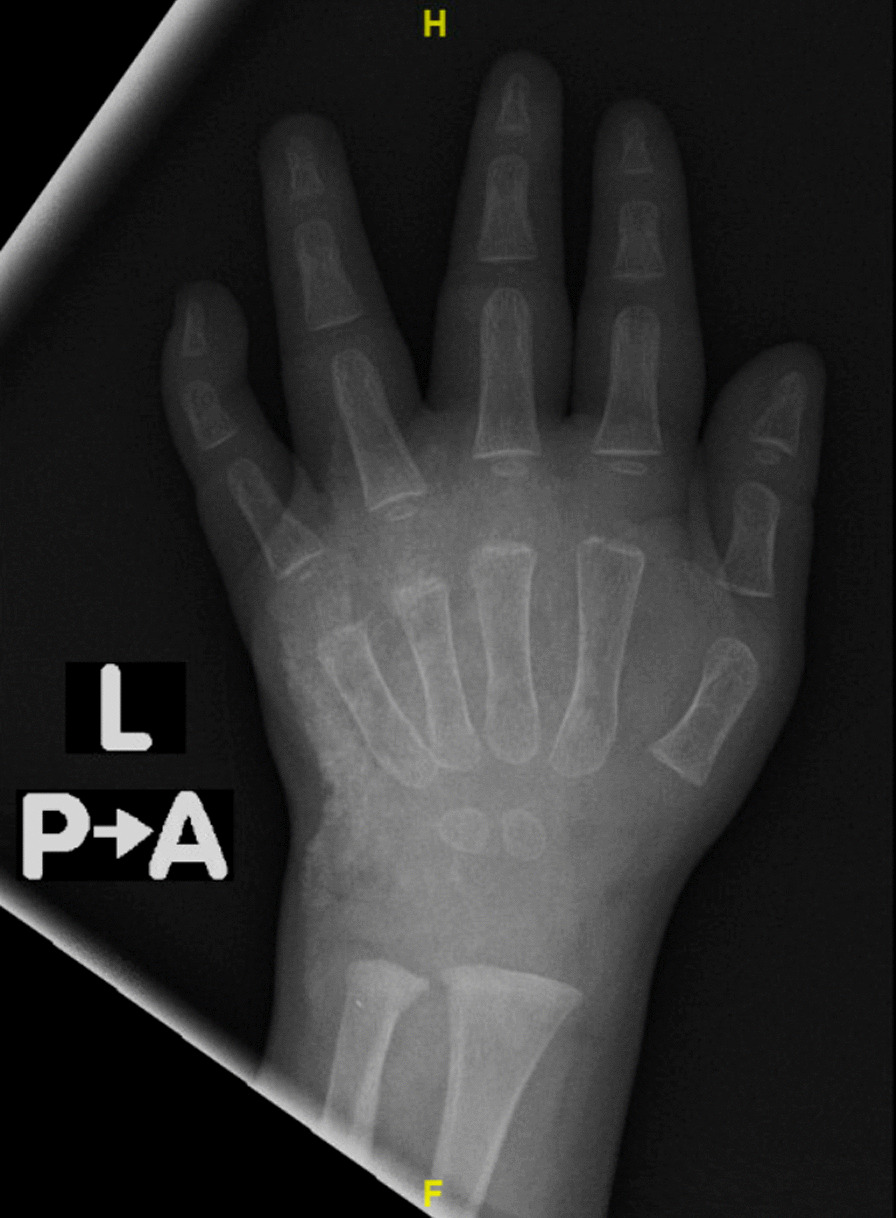
Subcutaneous calcification of the left hand

**Fig. 4:**
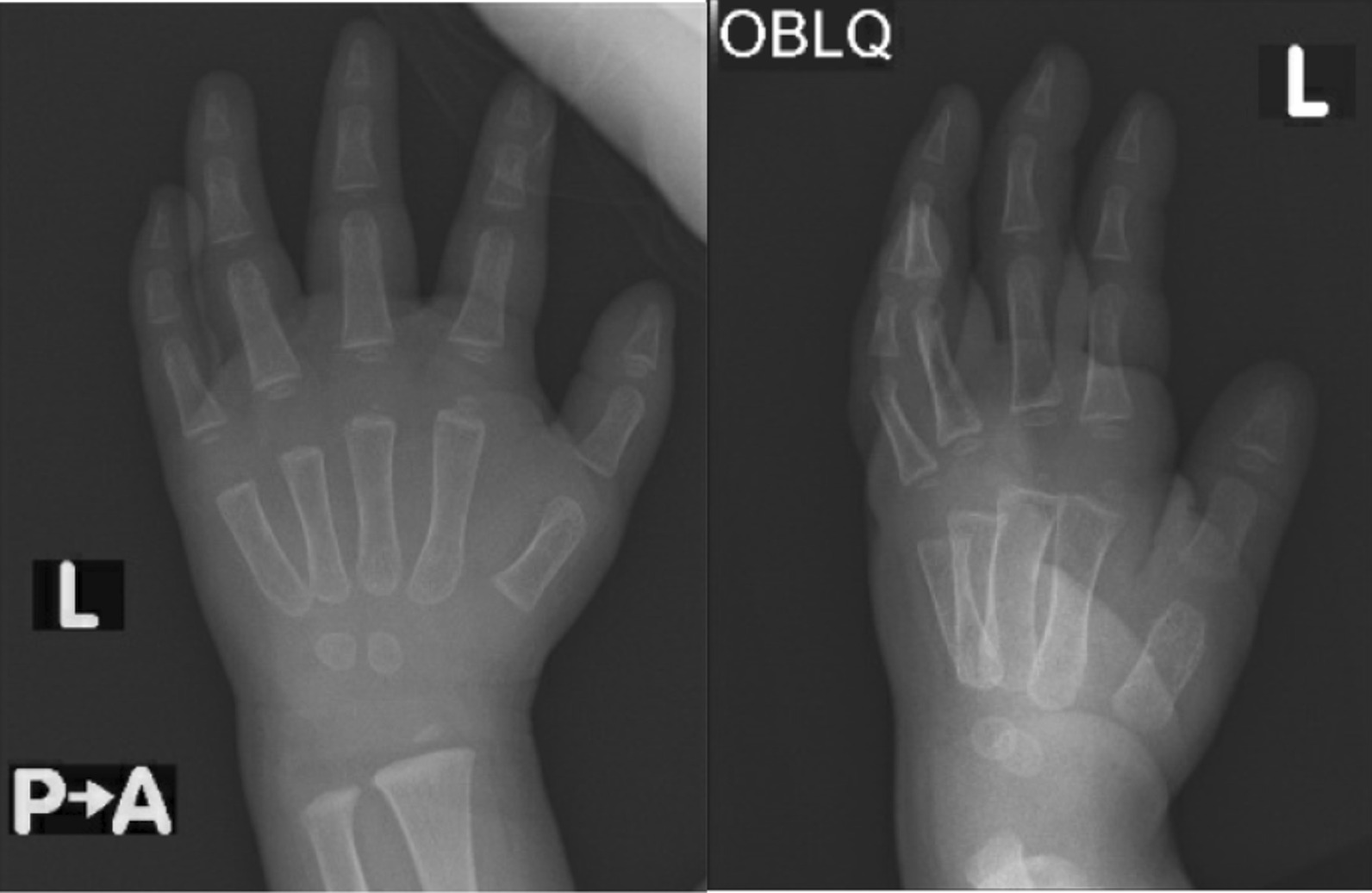
Complete disappearance of calcification

X-ray (Figs. 2 and 3) showed calcification from the intravenous calcium given earlier. A diagnosis of iatrogenic calcinosis cutis was made. He was managed conservatively with multiple follow-ups, and a left-hand X-ray was scheduled for after 2 months. Complete resolution of calcification happened within 2 months (Fig. 4).

## Discussion

The clinical and radiological diagnosis of our case suggested calcinosis cutis of the hand following the treatment of hypocalcemic seizure with calcium gluconate infusion.

Extravasation of calcium gluconate is a complication that is not uncommon, resulting in soft tissue and skin inflammation [3]. There are many causes of calcinosis cutis, including trauma, tumor, connective tissue disease, and high phosphate or calcium level [1]. On physical examination, bluish discoloration, redness, and mild restriction of the movement were noted in our case, which was initially thought to be cellulitis; however, the absence of fever warranted broadening the differential diagnosis. Most cases of iatrogenic calcinosis cutis are associated with a recent history of hospital admission [4]. Sawke *et al*. report a case with a history of administration of intravenous calcium, after which the patient developed subcutaneous nodule in the cubital fossa [4]. Pathophysiology of iatrogenic calcinosis cutis may be due to a transient increase in the concentration of calcium within the site of injection and associated tissue damage [4].

This leads to the formation of insoluble calcium deposits within the area of calcium infusion [4].

This complication could be reduced by checking the intravenous-line site frequently, to identify the issue earlier [3]. Other causes of iatrogenic calcinosis can include but are not limited to tumor lysis syndrome or frequent heel sticks in newborns [4]. Róbert *et al*. found that dystrophic calcinosis cutis was the most common type among other causes of calcinosis cutis, accounting for 70% of cases of calcinosis cutis. Autoimmune connective tissue disease was the leading cause of dystrophic type [5]. Another form of calcification is calciphylaxis, which is a pinnacular arteriolar calcification and ischemic cutaneous necrosis. It is more common in end-stage renal disease and may accompany high calcium and phosphate levels. Unlike calcinosis cutis, it is a life-threatening condition as it is associated with luminal stenosis and thrombosis [6]. Moreover, calcinosis cutis can develop in some rheumatological diseases, such as juvenile dermatomyositis and systemic sclerosis. When it appears with some rheumatologic diseases, it could be due to severity and delayed treatment of the disease or related to some genetic polymorphism [7, 8]. The exact type of calcinosis cutis should be accurately identified for proper treatment and management [4]. Diagnosis can be yield through fine-needle aspiration cytology, or imaging as in our case. Findings in fine-needle aspiration include whitish granular material [4]. It can be confirmed by applying hematoxylin and eosin or von Kossa silver stain to an alcohol-fixed smear in which it will show calcium deposits [1, 4]. Radiologic findings in plain radiograph may appear as multiple linear and stippled calcifications subcutaneously [7], as well as increased reuptake in bone scan suggesting heterotrophic ossification or myositis ossificans [9]. The diagnosis is frequently mistaken for cellulitis, osteomyelitis, arthritis, abscess, periostitis, and thrombophlebitis [10]. Diagnosis and management can be challenging; thus, it requires the collaboration of multiple health care providers to better achieve a good outcome and early management [1]. In a case report by Alsaif *et al.*, idiopathic unilateral calcinosis cutis was managed conservatively after excluding other potential causes for calcinosis cutis [2]. There was no change in the number of lesions nor a change in her health status in a follow-up [2]. In another case, an 11-year-old girl with osteogenic sarcoma developed iatrogenic calcinosis of the left wrist following treatment with calcium gluconate infusion and was managed conservatively, as in our reported case, with wound care only [11]. In contrast, one case of chronic hemodialysis developed iatrogenic calcinosis cutis following the administration of low molecular weight heparin (nadroparin calcium, Fraxiparin), and was successfully medically managed with sodium thiosulfate and aluminum hydroxide [12]. Another report concerns a 5‐year‐old boy with acute lymphoblastic leukemia who developed severe calcinosis cutis in the right forearm and hand, along with the left leg and foot after extravasation of calcium gluconate during treatment for tumor‐lysis syndrome after having hypocalcemia. Surgical debridement along with local wound care, hyperbaric oxygen therapy, and sodium thiosulfate infusion achieved complete healing of all lesions in 8 months with short discontinuation of chemotherapy [13]. In another case, an infant with severe asphyxia and persistent pulmonary hypertension received intravenous calcium gluconate therapy for hypocalcemia. At 5 weeks of age, multiple firm and indurated areas were found in the subcutaneous tissues of her trunk, arms, legs, and face, particularly in skin folds. She was managed conservatively by removing all exogenous calcium sources, and she underwent close follow-ups [14]. Moreover, an 11-year-old girl, who developed calcinosis cutis of both elbows and buttocks following trauma, was managed successfully through surgical excision of the calcified masses with no recurrence on follow-up [9]. Multiple investigations should be conducted to discover the specific cause of calcification in high-risk populations, such as assessments of parathyroid hormone, vitamin D, phosphate, calcium, and renal function to evaluate for chronic kidney disease [1]. An increase in serum calcium and phosphate following treatment with intravenous calcium places a patient at risk of iatrogenic calcinosis cutis [15]. Treatment options for calcinosis cutis include conservative, medical, and surgical management. Limited and small lesions are better treated with surgery, whereas those that are large and affecting generalized areas are good candidates for medical management [1, 15]. A retrospective study discussed different treatments applied to patients with calcinosis cutis with underlying autoimmune connective tissue disease, including medical and surgical treatments. Surgical treatment of symptomatic lesions and medical treatment with a calcium channel blocker (diltiazem) were found to be effective in some patients [8]. There is still no definite treatment for calcinosis cutis as there is no drug proven to be effective in clinical trials; fortunately, the course of calcinosis cutis is benign, and the mainstay treatment is only supportive therapy and observation. All therapies proposed were derived from data generally reported in single cases and only small case series [3].

Our case was managed conservatively by discontinuing any exogenous calcium with close monitoring and follow-ups, including hand X-rays at each visit, as well as assessment of calcium, parathyroid hormone, phosphate, and vitamin D levels, which all turned out to be normal.

## Conclusion

In conclusion, careful history-taking and examination are mandatory to achieving accurate diagnosis, by excluding other possible causes. Checking the intravenous-line site more frequently and considering dilution of the intravenous calcium may reduce the risk of extravasation and allow the issue to be recognized early, leading to a better outcome. Physicians should be aware of complications and side effects of any given treatment and have a high index of suspicion.

## Data Availability

The datasets supporting the conclusions of this article are included within the article with its cited references.
